# Efficacy and Safety of Lianhuaqingwen Capsules for the Prevention of Coronavirus Disease 2019: A Prospective Open-Label Controlled Trial

**DOI:** 10.1155/2021/7962630

**Published:** 2021-11-23

**Authors:** Xiaowei Gong, Boyun Yuan, Yadong Yuan, Fengju Li

**Affiliations:** ^1^Department of Respiratory and Critical Care Medicine, The Second Hospital of Hebei Medical University, Shijiazhuang, Hebei Province, China; ^2^Department of Medical Imaging, Hebei Provincial Corps Hospital of CPAPF, Shijiazhuang, Hebei Province, China

## Abstract

Coronavirus disease 2019 (COVID-19) has become a global pandemic. Community and close contact exposures continue to drive the COVID-19 pandemic. There is no confirmed effective treatment for suspected cases and close contacts. Lianhuaqingwen (LH) capsules, a repurposed Chinese herbal product that is currently on the market, have proven effective for influenza and COVID-19. To determine the safety and efficacy of LH capsules for the prevention of COVID-19, we conducted a prospective open-label controlled trial of LH capsules on subjects who had close contact with people infected with COVID-19. Subjects received LH capsules (4 capsules, three times daily) or the usual medical observation for 14 days. The primary endpoint was the rate of positive nucleic acid tests of nasal and pharyngeal swabs during the quarantine medical observation period. We included 1976 patients, including 1101 in the treatment group and 875 in the control group. The rate of positive nucleic acid tests in the treatment group was significantly lower than that in the control group (0.27% vs. 1.14%, respectively; mean difference: −0.87%; 95% CI: −1.83 to −0.13; *p*=0.0174) during the quarantine medical observation period (14 days). Among subjects with different close contact states, there was no significant difference in the rate of positive nucleic acid test results among close contacts in the treatment group and the control group (6.45% vs. 11.43%, respectively; *p*=0.6762). Among secondary close contacts, the rate of positive nucleic acid tests in the treatment group was significantly lower than that in the control group (0.09% vs. 0.71%, respectively; *p*=0.0485). No serious adverse events were reported. Taken together, and in light of the safety and effectiveness profiles, these results show that LH capsules can be considered to prevent the progression of COVID-19 after close contact with an infected person. This trial is registered with ChiCTR2100043012.

## 1. Introduction

Coronavirus disease 2019 (COVID-19), a novel acute respiratory infectious disease, has been a major global public health event since 2019 [1]. Through more than one year of active prevention, control, and treatment, the epidemic in China has been effectively controlled, with only local sporadic cases in some areas and a few imported cases. However, as the global epidemic is still spreading and is likely to persist for the foreseeable future, the risk of COVID-19 transmission and spread in China will continue to exist. As of the beginning of 2021, COVID-19 infections are returning to China. To date, there have been confirmed cases of COVID-19 and asymptomatic infections in several provinces and cities, including Hebei and Guangdong. At present, strengthening the prevention and control of nosocomial infection, reducing the occurrence of nosocomial infection as much as possible, and enabling the early identification and isolation of asymptomatic cases and confirmed cases are the top priorities for controlling the source of infection and reducing the incidence of disease [2].

To obtain epidemiological information, such as the incidence, exposure history, and contact history of COVID-19 cases, and to conduct effective screening, evaluation, and management of the close contacts of novel coronavirus pneumonia cases, the National Health Commission formulated the COVID-19 Prevention and Control Plan (Eighth Edition) [3]. Detailed instructions were provided for the management of close contacts of COVID-19 patients. These requirements included centralized isolation and medical observation, health monitoring, regular nucleic acid testing, centralized management of close contacts according to the medical institutions designated for treatment, and the reduction of retransmission of the virus by close contacts to the greatest possible extent.

Plant-derived natural products, known as herb formulas, are commonly used in traditional Chinese medicine (TCM) for disease prevention and treatment worldwide [4]. For example, Danshen, the dried root of *Salvia miltiorrhiza* Bunge, has been used to treat cardiovascular diseases and hepatitis as a heart and liver meridian herb [5]. A study showed that topical sesame oil was noninferior to diclofenac gel for the reduction of knee osteoarthritis pain and the improvement of some functional indicators [6]. Based on the experiences of the Chinese population, it is claimed that the integration of TCM with conventional therapies and care could be beneficial for the treatment and management of patients affected by COVID-19 [7]. According to Chinese studies, TCM showed acceptable results for controlling COVID-19 in up to 90% of patients. In TCM, the enrichment of qi through acupuncture and herbal therapy strengthens the body, which is essential for fighting disease [8].

Lianhuaqingwen (LH) capsules, a patented new drug used to treat cold and flu symptoms (national drug approval Z20040063), is the first Chinese patent medicine to enter FDA clinical trials for the treatment of influenza in China. Since the outbreak of COVID-19, this treatment has been widely used in endemic areas nationwide, including designated hospitals and mobile cabin hospitals in Hubei, and it has been used by more than 70 million people for epidemic prevention and control. Prospective, retrospective clinical and basic experimental studies on the treatment of COVID-19 with LH have been carried out in China and have confirmed that with routine therapy, LH can improve clinical symptoms such as fever, fatigue, and cough; ameliorate lung computerized tomography (CT) features of the disease; shorten the duration of symptoms and treatment; and improve the rate of clinical cure [9]. *In vitro* experiments also confirmed that the expression of viral particles was significantly reduced after LH treatment, and the overexpression of the inflammatory cytokine genes *TNF-α*, *IL-6*, *MCP-1*, and *IP-10* was significantly inhibited in a dose-dependent manner [10]. LH has been listed in the Chinese Health Commission's “Novel Coronavirus Infection Pneumonia Diagnosis and Treatment Program” (Trial 4th/5th/6th/7th/8th edition) among the recommended drugs for the medical observation period [3]. The Expert Consensus on the Diagnosis, Treatment, and Prevention of Childhood Novel Coronavirus Infection (1st/2nd edition) [11] also recommended LH treatment. At the same time, it has been listed as the recommended drug in the COVID-19 diagnosis and treatment plans of 20 provincial health commissions and the TCM administrations in Hubei, Beijing, Shanghai, and other provinces and has become the most recommended Chinese patented medicine, playing a pivotal role in the prevention and control of the COVID-19 epidemic in China, particularly during the medical observation period. The purpose of this study was to evaluate the preventive effects of LH capsules in close contacts of COVID-19 infection cases and to provide real-world evidence for clinical prophylaxis.

## 2. Materials and Methods

### 2.1. Study Oversight

In this prospective practical clinical trial, we enrolled 1976 close contacts of COVID-19 cases in Hebei Province. The research scheme was approved by the Ethics Committee of the Second Hospital of Hebei Medical University. The protocol was designed according to the Good Clinical Practice guidelines and The Declaration of Helsinki and was registered with the China Clinical Trial Registry website (http://www.chictr.org/cn/, no. ChiCTR2100043012). All patients signed written informed consent forms. For participants under 18 years of age, written informed consent was provided by their parents.

### 2.2. Patients

**Figure 1:**
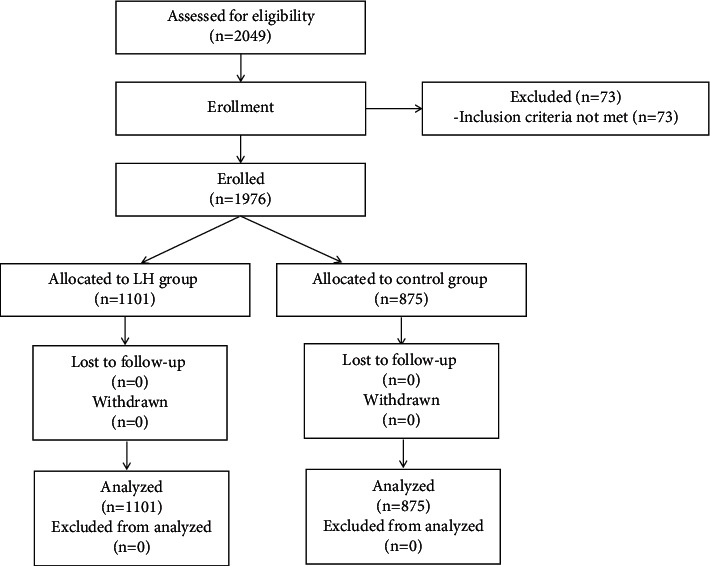
Study flow chart. FAS: full analysis set; PPS: per protocol set.

We recruited 1976 COVID-19 close contacts (including secondary close contacts) from February 2, 2021, to February 24, 2021 (Figure 1). The inclusion criteria were as follows: (1) close contacts of COVID-19 cases, confirmed by a flow survey; (2) at least 12 years of age, male or female; and (3) voluntarily signing a written informed consent form before the study began. The main exclusion criteria were as follows: (1) COVID-19 infection confirmed by etiological tests and clinical manifestations or signs; (2) an allergic constitution, such as an allergy history to two or more drugs or food or a known allergy to the ingredients of this drug; and (3) pregnancy or lactation.

Close contacts were defined as people who had close contact with suspected or confirmed COVID-19 cases within 2 days before the onset of symptoms but failed to take effective protective measures or people who had close contact with asymptomatic COVID-19 patients within 2 days before samples were taken and failed to take effective protective measures.

Secondary close contacts were defined as people who had close contact with close contacts, such as living with them, working in the same enclosed environment, and dining and entertaining together, and had not taken effective protective measures during the period between the close contacts' first contact with the confirmed or asymptomatic COVID-19 patients and the medical isolation of the close contacts.

### 2.3. Materials

The main components of LH are Forsythiae fructus (255 g), *Ephedrae* herba (honey-fried) (85 g), *Lonicerae japonicae* flos (255 g), Isatidis radix (255 g), *Dryopteris crassirhizomatis* rhizoma (255 g), menthol (7.5 g), gypsum fibrosum (255 g), Pogostemonis herba (85 g), *Rhodiolae crenulata* radix et rhizoma (85 g), *Houttuyniae herba* (255 g), Rhei radix et rhizoma (51 g), Semen armeniacae amarum (stir-baked) (85 g), and *Glycyrrhizae* radix et rhizoma (85 g), and the excipient is starch. These LH capsules were donated by Yiling Pharmaceutical, Inc., (Shijiazhuang, China), TCM Quasiword, Z20040063; product batch, A2008240; product specifications, 0.35 g per capsule.

The drug quality standard for LH complied with the provisions of Part I of the 2015 edition of the Chinese Pharmacopoeia.

### 2.4. Procedures

At present, the close contacts are concentrated in designated isolation points for isolated observation. For this study, different isolation points were selected. Some of the isolation points administered LH capsules (4 capsules, three times daily for 14 consecutive days) and were used as the experimental group, while other isolation points that provided only isolated medical observation were used as the control group. The general treatment of the patients in the two groups was based on the Coronavirus Pneumonia Diagnosis and Treatment Protocol (Trial 8 Edition) [3], which included ensuring rest, a relaxed mood, and adequate energy intake. Compliance with the use of the studied drug, clinical outcomes, concomitant drug use, and adverse events were recorded. Clinical symptoms and nucleic acid test results were assessed on the day of inclusion, day 7, and day 14.

### 2.5. Study Endpoints

The primary endpoint was the rate of positive nucleic acid tests performed on nasal and pharyngeal swabs during the quarantine medical observation period. The secondary endpoints included the following: the time of the occurrence of positive nucleic acid tests of nasal and pharyngeal swabs during the quarantine medical observation period; the proportion of asymptomatic people with positive nucleic acid tests; the proportion of mild, medium, and severe patients with positive nucleic acid tests; the date of onset during the quarantine medical observation period (i.e., the time when the individual began presenting clinical symptoms); and the classification of symptom severity during the medical observation period.

### 2.6. Safety Monitoring

There were no major reports of adverse events after the introduction of LH capsules [12]. In this study, we recorded the time, severity, duration, measurement, and consequences of adverse events. Using these data, we determined the association of adverse events with the use of the study drug.

### 2.7. Statistical Analysis

On February 29, 2020, the “World Health Organization COVID-19 Joint Expedition Report, China” [13] indicated the proportion of close contacts that subsequently became confirmed cases of COVID-19. Follow-up and medical observation of all identified close contacts indicated that 1–5% of close contacts were laboratory confirmed, with a 0.9% rate in Sichuan Province and a 4.8% rate in Guangdong Province. The independently released figure for Beijing was 5.8%. On March 3, 2020, the Shenzhen Centers for Disease Control and Prevention and the Johns Hopkins School of Public Health in the United States analyzed the data of 391 confirmed COVID-19 cases and 1286 close contacts in Shenzhen and found that less than 3% of close contacts became infected [14]. In addition, some studies found that the rate of positive nucleic acid detection among close contacts was as high as 9.49% [10].

According to a literature analysis, the rate of positive nucleic acid detection in close contacts of COVID-19 patients was close to 7%. In this study, the positive rate in the control group (nonintervention group) was conservatively estimated to be 7%, and it was assumed that the positive rate was 4% after the LH intervention. The ratio between groups was 1 : 1, and a 20% shedding rate was taken into account. The sample size was calculated as 2200 patients.

All statistical analyses were performed using SAS® 9.4 software (SAS Institute, Cary, NC). All of the patients were included in the full analysis set (FAS) after enrollment, while patients with major protocol deviation (PV) were removed from the per protocol set (PPS), and subjects who received one treatment for a safety evaluation were included in the safety data set (SS). All statistical tests were bilateral, and *p* < 0.05 was considered statistically significant. Count data are described as case numbers and composition ratios; measurement data are described as means, standard deviations, and maximum and minimum values; and nonnormally distributed data are reported as medians and 25th and 75th quantiles. General conditions between the two groups were compared using appropriate methods for the type of indicator. The *t*-test or Wilcoxon rank-sum test was used for the comparison of quantitative data between groups, the Chi-square test or accuracy probability test was used for data classification, and the Wilcoxon rank-sum test or CMH test was used for graded data. The incidence of adverse events was compared between groups using either the *χ*^2^ test or Fisher's exact probability method. The safety analysis was performed using the SS.

## 3. Results

### 3.1. Patient Characteristics

A total of 2049 subjects were screened for eligibility; 73 subjects could not be included. The main reason for noninclusion was not meeting the inclusion criteria, mainly because the subject was <12 years old (see Figure 1). A total of 1976 patients met the inclusion criteria, had good compliance and a mean medication duration of 14.0 days (95% CI: 12.0–15.0) if they were in the treatment group, and had no serious protocol violations. These patients were included in the FAS and SS (1101 cases in the treatment group and 875 cases in the control group). The research flow chart is shown in Figure 1.

At baseline, nearly 65% of the patients were under 45 years of age, and the gender distribution was near equal (50.35% male, 49.65% female). A total of 96.65% of the patients were secondary close contacts. The two groups were comparable in terms of demographic characteristics, close contact status, and drug combinations (Table 1).

### 3.2. Primary Endpoints

The rate of positive nucleic acid detection of COVID-19 from intranasal and pharyngeal swabs was significantly lower in the treatment group than in the control group (0.27% vs. 1.14%, respectively; mean difference: −0.87%; 95% CI: −1.83 to −0.13; *p*=0.0174, Table 2). All of the patients with positive nucleic acid tests shared a confined space with the contact case (8 cases) or were exposed through medical care (5 cases) (Table 3). Among subjects with different close contact statuses, there was no significant difference in the positive rate of nucleic acid detection from intranasal and pharyngeal swabs during the quarantine medical observation period (14 days) between the treatment group and the control group (6.45% vs. 11.43%, respectively; *p*=0.6762; Table 4). The positive rate of secondary close contacts in the treatment group was significantly lower than that of the control group (0.09% vs. 0.71%, respectively; *p*=0.0485; Table 4). LH had greater preventive effects on female patients than on male patients (*p*=0.014; Tables 5 and 6). There was no significant difference between the two groups in age or close contact modes other than those described above (Tables 2 and 7).

### 3.3. Secondary Endpoints

Of the 13 patients with positive nucleic acid test results, one was symptomatic and exhibited mild symptoms, while the rest were asymptomatically infected (Table 8). During the medical observation period, a total of 24 patients developed symptoms (Table 9), of which fever was the most common; LH significantly reduced the incidence of fever (*p* < 0.001). In addition, the treatment group had a significantly lower rate of symptom occurrence than the control group (99.36% vs. 98.06%, respectively; *p*=0.0084; Table 10), but there was no statistically significant difference in symptom severity between the two groups (*p*=0.1402; Table 11).

The time to the occurrence of a positive nucleic acid test was 4.67 days in the treatment group and 8.50 days in the control group, which was not significantly different (*p*=0.3078, Table 1).

### 3.4. Safety

The only adverse event was diarrhea (2 cases, both in the treatment group). No serious adverse events were reported.

## 4. Discussion

To our knowledge, this is the first clinical trial that demonstrates the safety and efficacy of LH capsules in subjects who have had close contact with confirmed COVID-19 patients. Overall, treatment with LH capsules for 14 days resulted in a significantly lower rate of positive nucleic acid tests from nasal and pharyngeal swabs during the quarantine medical observation period. In addition, LH capsules had a favorable safety profile for the prevention of COVID-19.

COVID-19 has the characteristics of strong infectivity, rapid and easy transmission, and general susceptibility among people. It is infectious during the incubation period. Based on the current epidemiological investigation, the incubation period is approximately 1.0–14.0 days, most often 3.0–7.0 days, and infectivity is strong within 5 days after onset [3]. Unlike SARS-CoV-1 and MERS-CoV, most cases of SARS-CoV-2 infection are mild and asymptomatic. Unrecognized cases of COVID-19 may account for approximately 60% of all infections [15]. Asymptomatic infected patients may be highly infectious during the incubation period [16]. Community and close contact exposures continue to drive the COVID-19 pandemic [17]. At present, there is no confirmed effective drug for suspected and close contacts of people with COVID-19, and home observation and supportive clinical treatment of symptoms are most often adopted [3] as an important means of identifying hidden infections and potential risk factors. Thus, China's epidemic prevention and control work is facing a considerable challenge. Effective prevention and control measures for close contacts and drug research have become the primary tasks of clinical and scientific research. Because drug and vaccine development can be laborious and time-consuming, the investigation of existing drugs for activity against COVID-19 infection represents one of the most feasible strategies for rapidly identifying effective treatments.

LH, a form of TCM, was used for the treatment of influenza during the H1N1 flu outbreak. In a study of prophylactic drugs among 20,553 close contacts and the people around them in Langfang, Hebei Province, the incidence rate of symptoms in the LH group was 1.2%, while that of participants who took other drugs was 6.8%, and that of those who did not use drugs was 8.8%, indicating that LH has a good prophylactic effect [18]. The LH has also shown good clinical efficacy for the treatment of COVID-19, the illness caused by SARS-CoV-2. LH was included in the Diagnosis and Treatment Programs for the 2019 New Coronavirus Pneumonia (from the fourth to eighth editions) formulated by the National Health Commission of China, which was published with the intention of preventing and treating viral influenza [19].

LH is composed of a variety of medicinal ingredients, such as honeysuckle, forsythia, *Ephedra sinica*, *Isatis indigotica*, and *Dryopteris crassirhizoma*. Researchers have determined that LH has a broad spectrum of activity against a variety of viruses, including H1N1 [20], H7N9 [21], Middle East respiratory syndrome (MERS) coronavirus [22], and SARS-CoV [23]. As a proprietary Chinese medicine, LH has been suggested to have therapeutic effects on SARS-CoV-2 patients in clinical, *in vitro*, and mouse models [9, 10, 24, 25]. Key components such as honeysuckle and forsythia can block the binding of SARS-CoV-2 with an angiotensin-converting enzyme [26]. Rhodiola can improve lung injury by inhibiting oxidative stress and apoptosis [27] and eliminating lung inflammation [28]. In addition, *Ephedra sinica* can effectively antagonize the binding of spiroprotein and angiotensin-converting enzyme [29], inhibit the excessive release of inflammatory mediators, and thus reduce lung injury [30]. Li et al. recently reported that LH could effectively inhibit the replication of SARS-CoV-2 in Vero E6 cells with an IC50 of 411.2 *μ*g/mL and can significantly reduce the expression of several proinflammatory cytokines (IL-6, TNF-*α*, and CCL-2/MCP-1). These data provide preliminary evidence of LH's ability to protect the lungs and indicate its potential as a treatment for COVID-19-related lung injury [10].

A previous study found that 6.3% of close contacts of COVID-19 patients were infected with SARS-CoV-2. Among the close contacts of asymptomatic carriers, 4.4% were infected [16, 31], and the majority were also asymptomatic carriers [32]. In our study, treatment with LH capsules for 14 days could effectively prevent SARS-CoV-2 infection, and the rate of positive nucleic acid detection was significantly lower in the treatment group than in the control group (0.27% vs. 1.14%, respectively). The positive rate of secondary close contacts was significantly lower in the treatment group than in the control group (0.09% vs. 0.71%, respectively). In addition, the LH group had significantly fewer symptoms than the control group, and LH significantly reduced the probability of fever in particular. All of these results indicate that LH can effectively prevent SARS-CoV-2 infection. Furthermore, no serious adverse events were reported, supporting the safety of LH capsules for COVID-19 treatment.

In July 2020, a community and close contact study of COVID-19-related exposures in symptomatic adults aged 18 years or older at 11 outpatient care facilities in the United States [17] suggested that compared to the control group, SARS-CoV-2-infected persons and their close contacts were more likely to have eaten at a restaurant (in any area designated by the restaurant, including indoors, on terraces, and outdoor seats), gone out to dinner (OR = 2.8, 95% CI = 1.9–4.3), or gone to bars/coffee shops (OR = 3.9, 95% CI = 1.5–10.1). During the 14 days prior to the onset, 71% of case patients and 74% of control participants reported always using a cloth mask or other type of mask in public. Forty-two percent of the patients reported close contact with one or more known COVID-19 patients, compared to 14% of the control group (*p* < 0.01), and most (51%) of the close contacts were family members. In this study, all close contacts with confirmed infection had a history of exposure to the same confined environment (8 cases) or had received medical care (5 cases). These data show that it is particularly important to take protective measures against the spread of COVID-19 in public places.

Our results suggest that LH capsules are effective for the prevention of SARS-CoV-2 infection in close contacts of COVID-19 patients. However, there are some limitations to the design of this study. Because of the urgency of the epidemic and the need for timely treatment, blinding methods were not implemented. The duration of treatment was based on the current incubation period, and further research is needed to determine whether extending the duration of treatment increases efficacy. An extended study is needed to thoroughly explore the preventive effects of LH capsules against SARS-CoV-2.

## 5. Conclusion

In summary, LH capsules conferred preventive effects on those exposed to COVID-19. In light of their efficacy and safety profile, LH capsules can be considered useful for the prevention of COVID-19 upon exposure. Future double-blind, prospective, randomized controlled trials are needed to fully evaluate the efficacy of LH capsules in a larger patient population.

## Figures and Tables

**Table 1:** Comparison of the demographic data and general conditions of the enrolled subjects.

Variable		LH group (N = 1101)	Control group (N = 875)	*p* value
Age, years (median (IQR))		38.34 (10.00–89.00)	37.74 (10.00–89.00)	0.4572
Age group	12–17	199 (18.07)	105 (12.00)	0.0082
	18–45	494 (44.87)	474 (54.17)	
	46–70	358 (32.52)	278 (31.77)	
	>70	50 (4.54)	18 (2.06)	
Gender	Male, N (%)	543 (49.32)	452 (51.66)	0.3018
Drug combination	Yes, N (%)	2 (0.18)	0 (0.00)	0.5063
Contact status	Close contacts, N (%)	31 (2.82)	35 (4.00)	0.1455
	Secondary close contacts, N (%)	1070 (97.18)	840 (96.00)	
Contact form	Living together, N (%)	157 (14.26)	29 (3.31)	<0.0001
	Shared the same confined space, N (%)	115 (10.45)	240 (27.43)	
	Dined together, N (%)	93 (8.45)	26 (2.97)	
	Daily conversation, N (%)	332 (30.15)	138 (15.77)	
	Rode in the same vehicle, N (%)	76 (6.90)	370 (2.63)	
	Live on the same block, N (%)	169 (15.35)	23 (2.63)	
	Via medical care, N (%)	29 (2.63)	45 (5.14)	
	Others, N (%)	130 (11.81)	4 (0.46)	
Positive conversion time of RNA detection, days (median (IQR))		4.67	8.50	0.3078

**Table 2:** Comparison of positive rates between the two groups.

Follow-up time point	Positive rate	*N* = 1976		*p* value
		LH group (N = 1101)	Control group (N = 875)	
Isolation period	Positive, N (%)	3 (0.27)	10 (1.14)	0.0174
Rate difference (LH group − control group) % (95% CI)			−0.87 (−1.83 to −0.13)	

**Table 3:** Positive rates of subjects with different contact modes.

Contact form	Positive rate	*N* = 1976		*p* value
		LH group (N = 1101)	Control group (N = 875)	
Living together, N (%)	Positive, N (%)	0 (0.00)	0 (0.00)	
Shared the same confined space, N (%)	Positive, N (%)	3 (0.27)	5 (0.57)	0.7173
Dined together, N (%)	Positive, N (%)	0 (0.00)	0 (0.00)	
Daily conversation, N (%)	Positive, N (%)	0 (0.00)	0 (0.00)	
Rode in the same vehicle, N (%)	Positive, N (%)	0 (0.00)	0 (0.00)	
Live on the same block, N (%)	Positive, N (%)	0 (0.00)	0 (0.00)	
Medical care, N (%)	Positive, N (%)	0 (0.00)	5 (0.57)	0.1496
Others, N (%)	Positive, N (%)	0 (0.00)	0 (0.00)	

**Table 4:** Comparison of positive rates of subjects with different close contact statuses.

Contact status	Positive rate	*N* = 1976		*p* value
		LH group	Control group	
Close contact	N	31	35	0.6762
	Positive, N (%)	2 (6.45)	4 (11.43)	

Secondary close contact	N	1070	840	0.0485
	Positive, N (%)	1 (0.09)	6 (0.71)	

**Table 5:** Comparison of demographic data and general conditions of subjects with a positive nucleic acid test.

Variables		LH group N = 3	Control group N = 10	*p* value
Age, years (median (IQR))		44.67 (41.00–48.00)	41.30 (27.00–63.00)	0.6726
Age group	12–45	2 (66.67)	6 (60.00)	1.0000
	46–70	1 (33.33)	4 (40.00)	
	>70	0 (0.00)	0 (0.00)	
Gender	Male, N (%)	3 (100.00)	1 (10.00)	0.0140
Drug combinations	Yes, N (%)	0 (0.00)	0 (0.00)	
Contact status	Close contacts, N (%)	2 (66.67)	4 (40.00)	0.5594
	Secondary close contacts, N (%)	1 (33.33)	6 (60.00)	
Contact form	Living together, N (%)	0 (0.00)	0 (0.00)	0.2308
	Shared the same confined space, N (%)	3 (100.00)	5 (50.00)	
	Dined together, N (%)	0 (0.00)	0 (0.00)	
	Daily conversation, N (%)	0 (0.00)	0 (0.00)	
	Rode in the same vehicle, N (%)	0 (0.00)	0 (0.00)	
	Live in the same village, N (%)	0 (0.00)	0 (0.00)	
	Via medical care, N (%)	0 (0.00)	5 (50.00)	
	Others, N (%)	0 (0.00)	0 (0.00)	

**Table 6:** Comparison of positive rates of subjects of different genders.

Follow-up time	Positive rate	*N* = 1976		*p* value
		LH group	Control group	
Male	N	543	452	0.6305
	Positive, N (%)	3 (0.55)	1 (0.22)	

Female	N	558	423	0.0005
	Positive, N (%)	0 (0.00)	9 (2.13)	

**Table 7:** Positive rates of subjects in different age groups.

Age	Positive rate	*N* = 1976		*p* value
		LH group	Control group	
≤45		693	579	0.1518
	Positive, N (%)	2 (0.18)	6 (0.69)	

<45		358	278	0.1736
≤70	Positive, N (%)	1 (0.09)	4 (0.46)	

>70		50	18	
	Positive, N (%)	0 (0.00)	0 (0.00)	

**Table 8:** Proportion of asymptomatically infected subjects with positive nucleic acid tests.

Follow-up timepoint	Symptomaticity	*N* = 13		*p* value
		LH group (*N* = 3)	Control group (*N* = 10)	
Isolation period	Asymptomatic infection, N (%)	3 (100.00)	9 (90.00)	1.0000
	Symptomatic infection, N (%)	0 (0.00)	1 (10.00)	

**Table 9:** Comparison of symptoms during the medical observation period.

Variables	*N* = 1976		*p* value
	LH group (*N* = 1101)	Control group (*N* = 875)	
Fever	2 (0.18)	15 (1.71)	<0.001
Pharyngodynia	0 (0.00)	1 (0.11)	0.443
Cough	1 (0.09)	0 (0.00)	1.000
Expectoration	0 (0.00)	1 (0.11)	0.443
Diarrhea	2 (0.18)	0 (0.00)	0.506
Nasal obstruction	1 (0.09)	0 (0.00)	1.000
Rhinorrhea	1 (0.09)	0 (0.00)	1.000

**Table 10:** Proportion of subjects with symptoms during the medical observation period.

Follow-up timepoint	Symptomaticity	*N* = 1976		*p* value
		LH group (*N* = 1101)	Control group (*N* = 875)	
Isolation period	Symptomatic, N (%)	7 (0.64)	17 (1.94)	0.0084
	Asymptomatic, N (%)	1094 (99.36)	858 (98.06)	

**Table 11:** Severity of symptoms during the medical observation period.

Follow-up timepoint	Classification of symptoms	*N* = 24		*p* value
		LH group (*N* = 7)	Control group (*N* = 17)	
Isolation period	Level 1, N (%)	6 (85.71)	9 (52.94)	0.1402
	Level 2, N (%)	1 (14.29)	7 (41.18)	
	Level 3, N (%)	0 (0.00)	1 (5.88)	

## Data Availability

The data used to support the findings of this study are available from the corresponding author upon reasonable request.
